# Is a Long Essay Always a Good Essay? The Effect of Text Length on Writing Assessment

**DOI:** 10.3389/fpsyg.2020.562462

**Published:** 2020-09-25

**Authors:** Johanna Fleckenstein, Jennifer Meyer, Thorben Jansen, Stefan Keller, Olaf Köller

**Affiliations:** ^1^Department of Educational Research and Educational Psychology, Leibniz Institute for Science and Mathematics Education, Kiel, Germany; ^2^Institute for Psychology of Learning and Instruction, Kiel University, Kiel, Germany; ^3^School of Education, Institute of Secondary Education, University of Applied Sciences and Arts Northwestern Switzerland, Brugg, Switzerland

**Keywords:** text length, writing assessment, text quality, judgment bias, English as a foreign language, human raters, pre-service teachers

## Abstract

The assessment of text quality is a transdisciplinary issue concerning the research areas of educational assessment, language technology, and classroom instruction. Text length has been found to strongly influence human judgment of text quality. The question of whether text length is a construct-relevant aspect of writing competence or a source of judgment bias has been discussed controversially. This paper used both a correlational and an experimental approach to investigate this question. Secondary analyses were performed on a large-scale dataset with highly trained raters, showing an effect of text length beyond language proficiency. Furthermore, an experimental study found that pre-service teachers tended to undervalue text length when compared to professional ratings. The findings are discussed with respect to the role of training and context in writing assessment.

## Introduction

Judgments of students’ writing are influenced by a variety of text characteristics, including text length. The relationship between such (superficial) aspects of written responses and the assessment of text quality has been a controversial issue in different areas of educational research. Both in the area of educational measurement and of language technology, text length has been shown to strongly influence text ratings by trained human raters as well as computer algorithms used to score texts automatically (Chodorow and Burstein, 2004; Powers, 2005; Kobrin et al., 2011; Guo et al., 2013). In the context of classroom language learning and instruction, studies have found effects of text length on teachers’ diagnostic judgments (e.g., grades; Marshall, 1967; Osnes, 1995; Birkel and Birkel, 2002; Pohlmann-Rother et al., 2016). In all these contexts, the underlying question is a similar one: Should text length be considered when judging students’ writing – or is it a source of judgment bias? The objective of this paper is to investigate to what degree text length is a construct-relevant aspect of writing competence, or to what extent it erroneously influences judgments.

Powers (2005) recommends both correlational and experimental approaches for establishing the relevance of response length in the evaluation of written responses: “the former for ruling out response length (and various other factors) as causes of response quality (by virtue of their lack of relationship) and the latter for establishing more definitive causal links” (p. 7). This paper draws on data from both recommended approaches: A correlational analysis of a large-scale dataset [MEWS; funded by the German Research Foundation (Grant Nr. CO 1513/12-1) and the Swiss National Science Foundation (Grant Nr. 100019L_162675)] based on expert text quality ratings on the one hand, and an experimental study with untrained pre-service teachers on the other. It thereby incorporates the measurement perspective with the classroom perspective. In the past, (language) assessment research has been conducted within different disciplines that rarely acknowledged each other. While some assessment issues are relevant for standardized testing in large-scale contexts only, others pertain to research on teaching and classroom instruction as well. Even though their assessments may serve different functions (e.g., formative vs. summative or low vs. high stakes), teachers need to be able to assess students’ performance accurately, just as well as professional raters in standardized texts. Thus, combining these different disciplinary angles and looking at the issue of text length from a transdisciplinary perspective can be an advantage for all the disciplines involved. Overall, this paper aims to present a comprehensive picture of the role of essay length in human and automated essay scoring, which ultimately amounts to a discussion of the elusive “gold standard” in writing assessment.

## Theoretical Background

Writing assessment is about identifying and evaluating features of a written response that indicate writing quality. Overall, previous research has demonstrated clear and consistent associations between linguistic features on the one hand, and writing quality and development on the other. In a recent literature review, Crossley (2020) showed that higher rated essays typically include more sophisticated lexical items, more complex syntactic features, and greater cohesion. Developing writers also show movements toward using more sophisticated words and more complex syntactic structures. The studies presented by Crossley (2020) provide strong indications that linguistic features in texts can afford important insights into writing quality and development. Whereas linguistic features are generally considered to be construct-relevant when it comes to assessing writing quality, there are other textual features whose relevance to the construct is debatable. The validity of the assessment of students’ competences is negatively affected by construct-irrelevant factors that influence judgments (Rezaei and Lovorn, 2010). This holds true for professional raters in the context of large-scale standardized writing assessment as well as for teacher judgments in classroom writing assessment (both formative or summative). Assigning scores to students’ written responses is a challenging task as different text-inherent factors influence the accuracy of the raters’ or teachers’ judgments (e.g., handwriting, spelling: Graham et al., 2011; length, lexical diversity: Wolfe et al., 2016). Depending on the construct to be assessed, the influence of these aspects can be considered judgment bias. One of the most relevant and well-researched text-inherent factors influencing human judgments is text length. Crossley (2020) points out that his review does “not consider text length as a linguistic feature while acknowledging that text length is likely the strongest predictor of writing development and quality.” Multiple studies have found a positive relationship between text length and human ratings of text quality, even when controlling for language proficiency (Chenoweth and Hayes, 2001; McCutchen et al., 2008; McNamara et al., 2015). It is still unclear, however, whether the relation between text length and human scores reflects a true relation between text length and text quality (appropriate heuristic assumption) or whether it stems from a bias in human judgments (judgment bias assumption). The former suggests that text length is a construct-relevant factor and that a certain length is needed to effectively develop a point of view on the issue presented in the essay prompt, and this is one of the aspects taken into account in the scoring (Kobrin et al., 2007; Quinlan et al., 2009). The latter claims that text length is either completely or partly irrelevant to the construct of writing proficiency and that the strong effect it has on human judgment can be considered a bias (Powers, 2005). In the context of large-scale writing assessment, prompt-based essay tasks are often used to measure students’ writing competence (Guo et al., 2013). These essays are typically scored by professionally trained raters. These human ratings have been shown to be strongly correlated with essay length, even if this criterion is not represented in the assessment rubric (Chodorow and Burstein, 2004; Kobrin et al., 2011). In a review of selected studies addressing the relation between length and quality of constructed responses, Powers (2005) showed that most studies found correlations within the range of *r* = 0.50 to *r* = 0.70. For example, he criticized the SAT essay for encouraging wordiness as longer essays tend to score higher. Kobrin et al. (2007) found the number of words to explain 39% of the variance in the SAT essay score. The authors argue that essay length is one of the aspects taken into account in the scoring as it takes a certain length to develop an argument. Similarly, Deane (2013) argues in favor of regarding writing fluency a construct-relevant factor (also see Shermis, 2014; McNamara et al., 2015). In an analytical rating of text quality, Hachmeister (2019) could showed that longer texts typically contain more cohesive devices, which has a positive impact on ratings of text quality. In the context of writing assessment in primary school, Pohlmann-Rother et al. (2016) found strong correlations between text length and holistic ratings of text quality (*r* = 0.62) as well as the semantic-pragmatic analytical dimension (*r* = 0.62). However, they found no meaningful relationship between text length and language mechanics (i.e., grammatical and orthographical correctness; *r* = 0.09).

Text length may be considered especially construct-relevant when it comes to writing in a foreign language. Because of the constraints of limited language knowledge, writing in a foreign language may be hampered because of the need to focus on language rather than content (Weigle, 2003). Silva (1993), in a review of differences between writing in a first and second language, found that writing in a second language tends to be “more constrained, more difficult, and less effective” (p. 668) than writing in a first language. The necessity of devoting cognitive resources to issues of language may mean that not as much attention can be given to higher order issues such as content or organization (for details of this debate, see Weigle, 2003, p. 36 f.). In that context, the ability of writing longer texts may be legitimately considered as indicative of higher competence in a foreign language, making text length a viable factor of assessment. For example, Ruegg and Sugiyama (2010) showed that the main predictors of the content score in English foreign language essays were first, organization and second, essay length.

The relevance of this issue has further increased as systems of automated essay scoring (AES) have become more widely used in writing assessment. These systems offer a promising way to complement human ratings in judging text quality (Deane, 2013). However, as the automated scoring algorithms are typically modeled after human ratings, they are also affected by human judgment bias. Moreover, it has been criticized that, at this point, automated scoring systems mainly count words when computing writing scores (Perelman, 2014). Chodorow and Burstein (2004), for example, showed that 53% of the variance in human ratings can be explained by automated scoring models that use only the number of words and the number of words squared as predictors. Ben-Simon and Bennett (2007) provided evidence from National Assessment of Educational Progress (NAEP) writing test data that standard, statistically created e-rater models weighed essay length even more strongly than human raters (also see Perelman, 2014).

Bejar (2011) suggests that a possible tendency to reward longer texts could be minimized through the training of raters with responses at each score level that vary in length. However, Barkaoui (2010) and Attali (2016) both compared the holistic scoring of experienced vs. novice raters and – contrary to expectations – found that the correlation between essay length and scores was slightly stronger for the experienced group. Thus, the question of whether professional experience and training counteract or even reinforce the tendency to overvalue text length in scoring remains open.

Compared to the amount of research on the role of essay length in human and automated scoring in large-scale high-stakes contexts, little attention has been paid to the relation of text length and quality in formative or summative assessment by teachers. This is surprising considering the relevance of the issue for teachers’ professional competence: In order to assess the quality of students’ writing, teachers must either configure various aspects of text quality in a holistic assessment or hold them apart in an analytic assessment. Thus, they need to have a concept of writing quality appropriate for the task and they need to be aware of the construct-relevant and -irrelevant criteria (cf. the lens model; Brunswik, 1955). To our knowledge, only two studies have investigated the effect of text length on holistic teacher judgments, both of which found that longer texts receive higher grades. Birkel and Birkel (2002) found significant main effects of text length (long, medium, short) and spelling errors (many, few) on holistic teacher judgments. Osnes (1995) reported effects of handwriting quality and text length on grades.

Whereas research on the text length effect on classroom writing assessment is scarce, a considerable body of research has investigated how other text characteristics influence teachers’ assessment of student texts. It is well-demonstrated, for example, that pre-service and experienced teachers assign lower grades to essays containing mechanical errors (Scannell and Marshall, 1966; Marshall, 1967; Cumming et al., 2002; Rezaei and Lovorn, 2010). Scannell and Marshall (1966) found that pre-service teachers’ judgments were affected by errors in punctuation, grammar and spelling, even though they were explicitly instructed to grade on content alone. More recently, Rezaei and Lovorn (2010) showed that high quality essays containing more structural, mechanical, spelling, and grammatical errors were assigned lower scores than texts without errors even in criteria relating solely to content. Teachers failed to distinguish between formal errors and the independent quality of content in a student essay. Similarly, Vögelin et al. (2018, 2019) found that lexical features and spelling influenced not only holistic teacher judgments of students’ writing in English as a second or foreign language, but also their assessment of other analytical criteria (e.g., grammar). Even though these studies do not consider text length as a potential source of bias, they do show that construct-irrelevant aspects influence judgments of teachers.

## This Research

Against this research background, it remains essential to investigate whether the relation between essay length and text quality represents a true relationship or a bias on the part of the rater or teacher (Wolfe et al., 2016). First, findings of correlational studies can give us an indication of the effect of text length on human ratings above and beyond language proficiency variables. Second, going beyond correlational findings, there is a need for experimental research that examines essay responses on the same topic differing only in length in order to establish causal relationships (Kobrin et al., 2007). The present research brings together both of these approaches.

This paper comprises two studies investigating the role of essay length in foreign language assessment using an interdisciplinary perspective including the fields of foreign language education, computer linguistics, educational research, and psychometrics. Study 1 presents a secondary analysis of a large-scale dataset with *N* = 2,722 upper secondary school students in Germany and Switzerland who wrote essays in response to “independent writing” prompts of the internet-based Test of English as a Foreign Language (TOEFL iBT). It investigates the question of how several indicators of students’ English proficiency (English grade, reading and listening comprehension, self-concept) are related to the length of their essays (word count). It further investigates whether or not essay length accounts for variance in text quality scores (expert ratings) even when controlling for English language proficiency and other variables (e.g., country, gender, cognitive ability). A weak relationship of proficiency and length as well as a large proportion of variance in text quality explained by length beyond proficiency would be in favor of the judgment bias assumption.

Study 2 focused on possible essay length bias in an experimental setting, investigating the effect of essay length on text quality ratings when there was (per design) no relation between essay length and text quality score. Essays from Study 1 were rated by *N* = 84 untrained pre-service teachers, using the same TOEFL iBT rubric as the expert raters. As text quality scores were held constant within all essay length conditions, any significant effect of essay length would indicate a judgment bias. Both studies are described in more detail in the following sections.

## Study 1

This study investigates the question of judgment bias assumption vs. appropriate heuristic assumption in a large-scale context with professional human raters. A weak relationship between text length and language proficiency would be indicative of the former assumption, whereas a strong relationship would support the latter. Moreover, if the impact of text length on human ratings was significant and substantial beyond language proficiency, this might indicate a bias on the part of the rater rather than an appropriate heuristic. Thus, Study 1 aims to answer the following research questions:

(1) How is essay length related to language proficiency?
(2) Does text length still account for variance in text quality when English language proficiency is statistically controlled for?

### Materials and Methods

#### Sample and Procedure

The sample consisted of *N* = 2,722 upper secondary students (11th grade; 58.1% female) in Germany (*n* = 894) and Switzerland (*n* = 1828) from the interdisciplinary and international research project *Measuring English Writing at Secondary Level* (MEWS; for an overview see Keller et al., 2020). The target population were students attending the academic track of general education grammar schools (ISCED level 3a) in the German federal state Schleswig-Holstein as well as in seven Swiss cantons (Aargau, Basel Stadt, Basel Land, Luzern, St. Gallen, Schwyz, Zurich). In a repeated-measures design, students were assessed at the beginning (T1: August/September 2016; *M*_*age*_ = 17.34; *SD*_*age*_ = 0.87) and at the end of the school year (T2: May/June 2017; *M*_*age*_ = 18.04; *SD*_*age*_ = 0.87). The students completed computer-based tests on writing, reading and listening skills, as well as general cognitive ability. Furthermore, they completed a questionnaire measuring background variables and individual characteristics.

#### Measures

##### Writing prompt

All students answered two independent and two integrated essay writing prompts of the internet-based Test of English as a Foreign Language (TOEFL iBT^®^) that is administered by the Educational Testing Service (ETS) in Princeton. The task instruction was as follows: “In the writing task below you will find a question on a controversial topic. Answer the question in an essay in English. List arguments and counter-arguments, explain them and finally make it clear what your own opinion on the topic is. Your text will be judged on different qualities. These include the presentation of your ideas, the organization of the essay and the linguistic quality and accuracy. You have 30 min to do this. Try to use all of this time as much as possible.” This task instruction was followed by the essay prompt. The maximum writing time was 30 min according to the official TOEFL iBT^®^ assessment procedure. The essays were scored by trained human raters on the TOEFL 6-point rating scale at ETS. In addition to two human ratings per essay, ETS also provided scores from their automated essay scoring system (e-rater^®^; Burstein et al., 2013). For a more detailed description of the scoring procedure and the writing prompts see Rupp et al. (2019) and Keller et al. (2020). For the purpose of this study, we selected the student responses to the TOEFL iBT independent writing prompt “Teachers,” which showed good measurement qualities (see Rupp et al., 2019). Taken together, data collections at T1 and T2 yielded *N* = 2,389 valid written responses to the following prompt: “A teacher’s ability to relate well with students is more important than excellent knowledge of the subject being taught.”

##### Text quality and length

The rating of text quality via human and machine scoring was done by ETS. All essays were scored by highly experienced human raters on the operational holistic TOEFL iBT rubric from 0 to 5 (Chodorow and Burstein, 2004). Essays were scored high if they were well-organized and individual ideas were well-developed, if they used specific examples and support to express learners’ opinion on the subject, and if the English language was used accurately to express learners’ ideas. Essays were assigned a score of 0 if they were written in another language, were generally incomprehensible, or if no text was entered.

Each essay received independent ratings by two trained human raters. If the two ratings showed a deviation of 1, the mean of the two scores was used; if they showed a deviation of 2 or more, a third rater (adjudicator) was consulted. Inter-rater agreement, as measured by quadratic weighted kappa (QWK), was satisfying for the prompt “Teachers” at both time points (QWK = 0.67; Hayes and Hatch, 1999; see Rupp et al., 2019 for further details). The mean text quality score was *M* = 3.35 (*SD* = 0.72).

Word count was used to measure the length of the essays. The number of words was calculated by the e-Rater scoring engine. The mean word count was *M* = 311.19 (*SD* = 81.91) and the number of words ranged from 41 to 727. We used the number of words rather than other measures of text length (e.g., number of letters) as it is the measure which is most frequently used in the literature: 9 out of 10 studies in the research review by Powers (2005) used word count as the criterion (also see Kobrin et al., 2007, 2011; Crossley and McNamara, 2009; Barkaoui, 2010; Attali, 2016; Wolfe et al., 2016; Wind et al., 2017). This approach ensures that our analyses can be compared with previous research.

##### English language proficiency and control variables

Proficiency was operationalized by a combination of different variables: English grade, English writing self-concept, reading and listening comprehension in English. The listening and reading skills were measured with a subset of items from the German National Assessment (Köller et al., 2010). The tasks require a detailed understanding of long, complex reading and listening texts including idiomatic expressions and different linguistic registers. The tests consisted of a total of 133 items for reading, and 118 items for listening that were administered in a multi-matrix-design. Each student was assessed with two rotated 15-min blocks per domain. Item parameters were estimated using longitudinal multidimensional two-parameter item response models in M*plus* version 8 (Muthén and Muthén, 1998–2012). Student abilities were estimated using 15 plausible values (PVs) per person. The PV reliabilities were 0.92 (T1) and 0.76 (T2) for reading comprehension, and 0.85 (T1) and 0.72 (T2) for listening comprehension. For a more detailed description of the scaling procedure see Köller et al. (2019).

General cognitive ability was assessed at T1 using the subtests on figural reasoning (N2; 25 items) and on verbal reasoning (V3; 20 items) of the Cognitive Ability Test (KFT 4–12 + R; Heller and Perleth, 2000). For each scale 15 PVs were drawn in a two-dimensional item response model. For the purpose of this study, the two PVs were combined to 15 overall PV scores with a reliability of 0.86.

The English writing self-concept was measured with a scale consisting of five items (e.g., “I have always been good at writing in English”; Eccles and Wigfield, 2002; Trautwein et al., 2012; α = 0.90). Furthermore, country (Germany = 0/Switzerland = 1), gender (male = 0/female = 1) and time of measurement (T1 = 0; T2 = 1) were used as control variables.

### Statistical Analyses

All analyses were conducted in M*plus* version 8 (Muthén and Muthén, 1998–2012) based on the 15PV data sets using robust maximum likelihood estimation to account for a hierarchical data structure (i.e., students clustered in classes; type = complex). Full-information maximum likelihood was used to estimate missing values in background variables. Due to the use of 15PVs, all analyses were run 15 times and then averaged (see Rubin, 1987).

Confirmatory factor analysis was used to specify a latent proficiency factor. All four proficiency variables showed substantial loadings in a single-factor measurement model (English grade: 0.67; writing self-concept: 0.73; reading comprehension: 0.42; listening comprehension: 0.51). As reading and listening comprehension were measured within the same assessment framework and could thus be expected to share mutual variance beyond the latent factor, their residuals were allowed to correlate. The analyses yielded an acceptable model fit: χ^2^(1) = 3.65, *p* = 0.06; CFI = 0.998, RMSEA = 0.031, SRMR = 0.006.

The relationship between text length and other independent variables was explored with correlational analysis. Multiple regression analysis with latent and manifest predictors was used to investigate the relations between text length, proficiency, and text quality.

### Results

The correlation of the latent proficiency factor and text length (word count) was moderately positive: *r* = 0.36, *p* < 0.01. This indicates that more proficient students tended to write longer texts. Significant correlations with other variables showed that students tended to write longer texts at T1 (*r* = -0.08, *p* < 0.01), girls wrote longer texts than boys (*r* = 0.11, *p* < 0.01), and higher cognitive ability was associated with longer texts (*r* = 0.07, *p* < 0.01). However, all of these correlations were very weak as a general rule. The association of country and text length was not statistically significant (*r* = -0.06, *p* = 0.10).

Table 1 presents the results of the multiple linear regression of text quality on text length, proficiency and control variables. The analysis showed that proficiency and the covariates alone explained 38 percent of the variance in text quality ratings, with the latent proficiency factor being by far the strongest predictor (Model 1). The effect of text length on the text quality score was equally strong when including the control variables but not proficiency in the model (Model 2). When both the latent proficiency factor and text length were entered into the regression model (Model 3), the coefficient of text length was reduced but remained significant and substantial, explaining an additional 24% of the variance (ΔR^2^ = 0.24 from Model 1 to Model 3). Thus, text length had an incremental effect on text quality beyond a latent English language proficiency factor.

**TABLE 1:** Linear regression of text quality on text length, English language proficiency, and control variables: standardized regression coefficients (β) and standard errors (SE).

**Predictors/R^2^**	**β (*SE*)**		
	**Model 1**	**Model 2**	**Model 3**
Text length		0.59 (0.02)**	0.41 (0.02)**
English language proficiency	0.65 (0.03)**		0.56 (0.03)**
Country	0.07 (0.02)**	0.14 (0.02)**	0.12 (0.02)**
Gender	0.07 (0.02)**	0.05 (0.02)**	0.02 (0.02)
Cognitive ability	−0.14 (0.03)**	0.14 (0.02)**	−0.08 (0.03)*
Time (T1/T2)	0.03 (0.02)	0.08 (0.02)**	0.06 (0.02)**
*R*^2^	0.38 (0.04)**	0.40 (0.02)**	0.62 (0.02)**

*^∗∗^p < 0.01; ^∗^p < 0.05.*

### Discussion

Study 1 approached the issue of text length by operationalizing the construct of English language proficiency and investigating how it affects the relationship of text length and text quality. This can give us an idea of how text length may influence human judgments even though it is not considered relevant to the construct of writing competence. These secondary analyses of an existing large-scale dataset yielded two central findings: First, text length was only moderately associated with language proficiency. Second, text length strongly influenced writing performance beyond proficiency. Thus, it had an impact on the assigned score that was not captured by the construct of proficiency. These findings could be interpreted in favor of the judgment bias assumption as text length may include both construct-irrelevant and construct-relevant information.

The strengths of this study were the large sample of essays on the same topic and the vast amount of background information that was collected on the student writers (proficiency and control variables). However, there were three major limitations: First, the proficiency construct captured different aspects of English language competence (reading and listening comprehension, writing self-concept, grade), but that operationalization was not comprehensive. Thus, the additional variance explained by text length may still have been due to other aspects that could not be included in the analyses as they were not in the data. Further research with a similar design (primary or secondary analyses) should use additional variables such as grammar/vocabulary knowledge or writing performance in the first language.

The second limitation was the correlational design, which does not allow a causal investigation of the effect of text length on text quality ratings. Drawing inferences which are causal in nature would require an experimental environment in which, for example, text quality is kept constant for texts of different lengths. For that reason, Study 2 was conducted exactly in such a research design.

Last but not least, the question of transferability of these findings remains open. Going beyond standardized large-scale assessment, interdisciplinary research requires us to look at the issue from different perspectives. Findings pertaining to professional raters may not be transferable to teachers, who are required to assess students’ writing in a classroom context. Thus, Study 2 drew on a sample of preservice English teachers and took a closer look at how their ratings were impacted by text length.

## Study 2

### Research Questions

In Study 2, we investigated the judgment bias assumption vs. the appropriate heuristic assumption of preservice teachers. As recommended by Powers (2005), we conducted an experimental study in addition to the correlational design used in Study 1. As text quality scores were held constant within all essay length conditions, any significant effect of essay length would be in favor of the judgment bias assumption. The objective of this study was to answer the following research questions:

(1) How do ratings of pre-service teachers correspond to expert ratings?
(2) Is there an effect of text length on the text quality ratings of preservice English teachers, when there is (per design) no relation between text length and text quality (main effect)?
(3) Does the effect differ for different levels of writing performance (interaction effect)?

### Materials and Methods

#### Participants and Procedure

The experiment was conducted with *N* = 84 pre-service teachers (*M*_*Age*_ = 23 years; 80% female), currently enrolled in a higher education teacher training program at a university in Northern Germany. They had no prior rating experience of this type of learner texts. The experiment was administered with the Student Inventory ASSET (Jansen et al., 2019), an online tool to assess students’ texts within an experimental environment. Participants were asked to rate essays from the MEWS project (see Study 1) on the holistic rubric used by the human raters at ETS (0–5; https://www.ets.org/s/toefl/pdf/toefl_writing_rubrics.pdf). Every participant had to rate 9 out of 45 essays in randomized order, representing all possible combinations of text quality and text length. Before the rating process began, participants were given information about essay writing in the context of the MEWS study (school type; school year; students’ average age; instructional text) and they were presented the TOEFL writing rubric as the basis for their judgments. They had 15 min to get an overview of all nine texts before they were asked to rate each text on the rubric. Throughout the rating process, they were allowed to highlight parts of the texts.

The operationalization of text quality and text length as categorical variables as well as the procedure of selecting an appropriate essay sample for the study is explained in the following.

#### Text Length and Text Quality

The essays used in the experiment were selected on the basis of the following procedure, which took both text quality and text length as independent variables into account. The first independent variable of the essay (overall text quality) was operationalized via scores assigned by two trained human raters from ETS on a holistic six-point scale (0–5; see Study 1 and Appendix A). In order to measure the variable as precisely as possible, we only included essays for which both human raters had assigned the same score, resulting in a sample of *N* = 1,333 essays. As a result, three gradations of text quality were considered in the current study: lower quality (score 2), medium quality (score 3) and higher quality (score 4). The corpus included only few texts (10.4%) with the extreme scores of 0, 1, and 5; these were therefore excluded from the essay pool. We thus realized a 3 × 3 factorial within-subjects design. The second independent variable text length was measured via the word count of the essays, calculated by the e-rater (c) scoring engine. As with text quality, this variable was subdivided in three levels: rather short texts (s), medium-length texts (m), and long texts (l). All available texts were analyzed regarding their word count distribution. Severe outliers were excluded. The remaining *N* = 1308 essays were split in three even groups: the lower (=261 words), middle (262–318 words) and upper third (=319 words). Table 2 shows the distribution of essays for the resulting combinations of text length and text score.

**TABLE 2:** Distribution of essays in the sample contingent on text quality and text length groupings.

**Text quality**	**Text length**			
	**Short (s)**	**Medium (m)**	**Long (l)**	**Total**
Low (2)	*n* = 147	*n* = 33	*n* = 15	*n* = 195
Medium (3)	*n* = 260	*n* = 299	*n* = 204	*n* = 763
High (4)	*n* = 22	*n* = 110	*n* = 218	*n* = 350
Total	*n* = 429	*n* = 442	*n* = 437	*N* = 1,308

*Number of essays excluding text quality scores 0, 1, and 5 as well as severe outliers concerning word count.*

#### Selection of Essays

For each text length group (s, m, and l), the mean word count across all three score groups was calculated. Then, the score group (2, 3, or 4) with the smallest number of essays in a text length group was taken as reference (e.g., *n* = 22 short texts of high quality or *n* = 15 long texts of low quality). Within each text length group, the five essays being – word count-wise – closest to the mean of the reference were chosen for the study. This was possible with mostly no or only minor deviations. In case of multiple possible matches, the essay was selected at random. This selection procedure resulted in a total sample of 45 essays, with five essays for each combination of score group (2, 3, 4) and length group (s, m, l).

### Results

**FIGURE 1:**
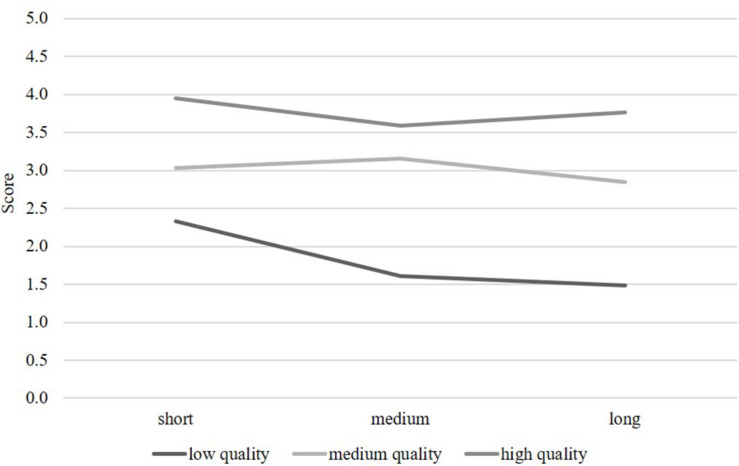
Visualization of the interaction between text length and text quality.

A repeated-measures ANOVA with two independent variables (text quality and text length) was conducted to test the two main effects and their interaction on participants’ ratings (see Table 3). Essay ratings were treated as a within-subject factor, accounting for dependencies of the ratings nested within raters. The main effect of text quality scores on participants’ ratings showed significant differences between the three text quality conditions (*low*, *medium*, *high*) that corresponded to expert ratings; *F*(2, 82) = 209.04, *p* < 0.001, *d* = 4.52. There was also a significant main effect for the three essay length conditions (*short*, *medium*, *long*); *F*(2, 82) = 9.14, *p* < 0.001, *d* = 0.94. Contrary to expectations, essay length was negatively related to participants’ ratings, meaning that shorter texts received higher scores than longer texts. The interaction of text quality and text length also had a significant effect; *F*(4, 80) = 3.93, *p* < 0.01, *d* = 0.89. *Post-hoc* tests revealed that texts of low quality were especially impacted by essay length in a negative way (see Figure 1).

**TABLE 3:** Participants’ ratings of text quality: means (M) and standard deviations (SD).

**Text quality**	**Text length**			
	**Short (s)**	**Medium (m)**	**Long (l)**	**Row total**
	***M* (*SD*)**	***M* (*SD*)**	***M* (SD)**	***M* (*SD*)**
Low (2)	2.33 (1.38)^*a*^	1.61 (0.92)^*b*^	1.49 (1.17)^*b*^	1.81 (1.23)
Medium (3)	3.04 (0.96)^*a*^	3.15 (1.41)^*a*^	2.85 (1.23)^*a*^	3.01 (1.22)
High (4)	3.95 (1.10)^*a*^	3.58 (1.12)^*b*^	3.76 (0.94)^*b*^	3.77 (1.06)
**Column total**	3.11 (1.33)^*a*^	2.78 (1.44)^*b*^	2.70 (1.46)^*b*^	

*Different superscript letters within a row indicate significant mean differences (p < 0.05).*

### Discussion

The experiment conducted in Study 2 found a very strong significant main effect for text quality, indicating a high correspondence of pre-service teachers’ ratings with the expert ratings of text quality. The main effect of text length was also significant, but was qualified by a significant interaction effect text quality *x* text length, indicating that low quality texts were rated even more negative the longer they were. This negative effect of text length was contrary to expectations: The pre-service teachers generally tended to assign higher scores to shorter texts. Thus, they seemed to value shorter texts over longer texts. However, this was mainly true for texts of low quality.

These findings were surprising against the research background that would suggest that longer texts are typically associated with higher scores of text quality, particularly in the context of second language writing. Therefore, it is even more important to discuss the limitations of the design before interpreting the results: First, the sample included relatively inexperienced pre-service teachers. Further research is needed to show whether these findings are transferable to in-service teachers with reasonable experience in judging students’ writing. Moreover, further studies could use assessment rubrics that teachers are more familiar with, such as the CEFR (Council of Europe, 2001; also see Fleckenstein et al., 2020). Second, the selection process of essays may have reduced the ecological validity of the experiment. As there were only few long texts of low quality and few short texts of high quality in the actual sample (see Table 2), the selection of texts in the experimental design was – to some degree – artificial. This could also have influenced the frame of reference for the pre-service teachers as the distribution of the nine texts was different from what one would find naturally in an EFL classroom. Third, the most important limitation of this study is the question of the reference norm, a point which applies to studies of writing assessment in general. In our study, writing quality was operationalized using expert ratings, which have been shown to be influenced by text length in many investigations as well as in Study 1. If the expert ratings are biased themselves, the findings of this study may also be interpreted as pre-service teachers (unlike expert raters) not showing a text length bias at all: shorter texts should receive higher scores than longer ones if the quality assigned by the expert raters is held constant. We discuss these issues concerning the reference norm in more detail in the next section.

All three limitations may have affected ratings in a way that could have reinforced a negative effect of text length on text quality ratings. However, as research on the effect of text length on teachers’ judgments is scarce, we should consider the possibility that the effect is actually different from the (positive) one typically found for professional human raters. There are a number of reasons to assume differences in the rating processes that are discussed in more detail in the following section. Furthermore, we will discuss what this means in terms of the validity of the gold standard in writing assessment.

## General Discussion

Combining the results of both studies, we have reason to assume that (a) text length induces judgment bias and (b) the effect of text length largely depends on the rater and/or the rating context. More specifically, the findings of the two studies can be summarized as follows: Professional human raters tend to reward longer texts beyond the relationship of text length and proficiency. Compared to this standard, inexperienced EFL teachers tend to undervalue text length, meaning that they sanction longer texts especially when text quality is low. This in turn may be based on an implicit expectation deeply ingrained in the minds of many EFL teachers: that writing in a foreign language is primarily about avoiding mistakes, and that longer texts typically contain more of them than shorter ones (Keller, 2016). Preservice teachers might be particularly afflicted with this view of writing as they would have experienced it as learners up-close and personal, not too long ago. Both findings point toward the judgment bias assumption, but with opposite directions. These seemingly contradictory findings lead to interesting and novel research questions – both in the field of standardized writing assessment and in the field of teachers’ diagnostic competence.

Only if we take professional human ratings as reliable benchmark scores can we infer that teachers’ ratings are biased (in a negative way). If we consider professional human ratings to be biased themselves (in a positive way), then the preservice teachers’ judgments might appear to be unbiased. However, it would be implausible to assume that inexperienced teachers’ judgments are less biased than those of highly trained expert raters. Even if professional human ratings are flawed themselves, they are the best possible measure of writing quality, serving as a reference even for NLP tools (Crossley, 2020). It thus makes much more sense to consider the positive impact of text length on professional human ratings – at least to a degree – an appropriate heuristic. This means that teachers’ judgments would generally benefit from applying the same heuristic when assessing students’ writing, as long as it does not become a bias.

In his literature review, Crossley (2020) sees the nature of the writing task to be among the central limitations when it comes to generalizing findings in the context of writing assessment. Written responses to standardized tests (such as the TOEFL) may produce linguistic features that differ from writing samples produced in the classroom or in other, more authentic writing environments. Moreover, linguistic differences may also occur depending on a writing sample being timed or untimed. Timed samples provide fewer opportunities for planning, revising, and development of ideas as compared to untimed samples, where students are more likely to plan, reflect, and revise their writing. These differences may surface in timed writing in such a way that it would be less cohesive and less complex both lexically and syntactically.

In the present research, such differences may account for the finding that pre-service teachers undervalue text length compared to professional raters. Even though the participants in Study 2 were informed about the context in which the writing samples were collected, they may have underestimated the challenges of a timed writing task in an unfamiliar format. In the context of their own classrooms, students rarely have strict time limitations when working on complex writing tasks. If they do, in an exam consisting of an argumentative essay, for example, it is usually closer to 90 min than to 30 min (at least in the case of the German pre-service teachers who participated in this study). Thus, text length may not be a good indicator of writing quality in the classroom. On the contrary, professional raters may value length as a construct-relevant feature of writing quality in a timed task, for example as an indicator of writing fluency (see Peng et al., 2020).

Furthermore, text length as a criterion of quality cannot be generalized over different text types at random. The genres which are taught in EFL courses, or assessed in EFL exams, differ considerably with respect to expected length. In five paragraph essays, for example, developing an argument requires a certain scope and attention to detail, so that text length is a highly salient feature for overall text quality. The same might not be true for e-mail writing, a genre frequently taught in EFL classrooms (Fleckenstein et al., in press). E-mails are usually expected to be concise and to the point, so that longer texts might seem prolix, or rambling. Such task-specific demands need to be taken into account when it comes to interpreting our findings. The professional raters employed in our study were schooled extensively for rating five-paragraph essays, which included a keen appreciation of text length as a salient criterion of text quality. The same might not be said of classroom teachers, who encounter a much wider range of genres in their everyday teaching and might therefore be less inclined to consider text length as a relevant feature. Further research should consider different writing tasks in order to investigate whether text length is particularly important to the genre of the argumentative essay.

Our results underscore the importance of considering whether or not text length should be taken into account for different contexts of writing assessment. This holds true for classroom assessment, where teachers should make their expectations regarding text length explicit, as well as future studies with professional raters. Crossley (2020) draws attention to the transdisciplinary perspective of the field as a source for complications: “The complications arise from the interdisciplinary nature of this type of research which often combines writing, linguistics, statistics, and computer science fields. With so many fields involved, it is often easy to overlook confounding factors” (p. 428). The present research shows how the answer to one and the same research question – How does text length influence human judgment? – can be very different from different perspectives and within different areas of educational research. Depending on the population (professional raters vs. pre-service teachers) and the methodology (correlational analysis vs. experimental design), our findings illustrate a broad range of possible investigations and outcomes. Thus, it is a paramount example of why interdisciplinary research in education is not only desirable but imperative. Without an interdisciplinary approach, our view of the text length effect would be uni-dimensional and fragmentary. Only the combination of different perspectives and methods can live up to the demands of a complex issue such as writing assessment, identify research gaps, and challenge research traditions. Further research is needed to investigate the determinants of the strength and the direction of the bias. It is necessary to take a closer look at the rating processes of (untrained) teachers and (trained) raters, respectively, in order to investigate similarities and differences. Research pertaining to judgment heuristics/biases can be relevant for both teacher and rater training. However, the individual concerns and characteristics of the two groups need to be taken into account. This could be done, for example, by directly comparing the two groups in an experimental study. Both in teacher education and in text assessment studies, we should have a vigorous discussion about how appropriate heuristics of expert raters can find their way into the training of novice teachers and inexperienced raters in an effort to reduce judgement bias.

## Data Availability Statement

The raw data supporting the conclusions of this article will be made available by the authors, without undue reservation, to any qualified researcher.

## Ethics Statement

The studies involving human participants were reviewed and approved by the Ministry of Education, Science and Cultural Affairs of the German federal state Schleswig-Holstein. Written informed consent to participate in this study was provided by the participants’ legal guardian/next of kin.

## Author Contributions

JF analyzed the data and wrote the manuscript. TJ and JM collected the experimental data for Study 2 and supported the data analysis. SK and OK provided the dataset for Study 1. TJ, JM, SK, and OK provided feedback on the manuscript. All authors contributed to the article and approved the submitted version.

## Conflict of Interest

The authors declare that the research was conducted in the absence of any commercial or financial relationships that could be construed as a potential conflict of interest.
